# Elevated air movement enhances stomatal sensitivity to abscisic acid in leaves developed at high relative air humidity

**DOI:** 10.3389/fpls.2015.00383

**Published:** 2015-05-28

**Authors:** Dália R. A. Carvalho, Sissel Torre, Dimitrios Kraniotis, Domingos P. F. Almeida, Ep Heuvelink, Susana M. P. Carvalho

**Affiliations:** ^1^Centro de Biotecnologia e Química Fina – Laboratório Associado, Escola Superior de Biotecnologia, Universidade Católica PortuguesaPorto, Portugal; ^2^Department of Plant Sciences, Norwegian University of Life SciencesÅs, Norway; ^3^Department of Mathematical Sciences and Technology, Norwegian University of Life SciencesÅs, Norway; ^4^Instituto Superior de Agronomia, Universidade de LisboaLisboa, Portugal; ^5^Horticulture and Product Physiology Group, Department of Plant Sciences, Wageningen UniversityWageningen, The Netherlands; ^6^Faculty of Sciences, University of PortoPorto, Portugal

**Keywords:** abscisic acid, relative air humidity, *Rosa* × *hybrida*, stomatal anatomy, stomatal function, vapor pressure deficit, wind speed

## Abstract

High relative air humidity (RH ≥ 85%) during growth leads to stomata malfunctioning, resulting in water stress when plants are transferred to conditions of high evaporative demand. In this study, we hypothesized that an elevated air movement (MOV) 24 h per day, during the whole period of leaf development would increase abscisic acid concentration ([ABA]) enhancing stomatal functioning. Pot rose ‘Toril’ was grown at moderate (61%) or high (92%) RH combined with a continuous low (0.08 m s^-1^) or high (0.92 m s^-1^) MOV. High MOV reduced stomatal pore length and aperture in plants developed at high RH. Moreover, stomatal function improved when high MOV-treated plants were subjected to leaflet desiccation and ABA feeding. Endogenous concentration of ABA and its metabolites in the leaves was reduced by 35% in high RH, but contrary to our hypothesis this concentration was not significantly affected by high MOV. Interestingly, in detached leaflets grown at high RH, high MOV increased stomatal sensitivity to ABA since the amount of exogenous ABA required to decrease the transpiration rate was significantly reduced. This is the first study to show that high MOV increases stomatal functionality in leaves developed at high RH by reducing the stomatal pore length and aperture and enhancing stomatal sensitivity to ABA rather than increasing leaf [ABA].

## Introduction

Stomatal opening and closing are influenced by environmental factors such as light, temperature, CO_2_ concentration, drought, RH and their interactions (Tallman, 2004; Reynolds-Henne et al. 2010; Feller and Vaseva, 2014). Long-term high RH (i.e., RH ≥ 85% throughout leaf ontogeny) is regarded as the environmental factor that mostly disturbs the capacity of stomata to close in response to water stress and darkness, leading to uncontrolled water loss (Torre et al., 2003; Rezaei Nejad and van Meeteren, 2005; In et al., 2006; Fanourakis et al., 2012). Recent studies have shown that although stomatal anatomy and density do contribute *per se* to the increased water loss in leaves developed at high RH, stomatal physiology is the major cause for this negative water balance (Fanourakis et al., 2013; Aliniaeifard et al., 2014). However, the reasons why stomata fail to close fully during water stress periods in plants grown under long-term high RH remain unclear.

Stomatal movement is to a large extent regulated by [ABA]. Lower [ABA], associated with higher stomatal aperture during growth, has been measured in leaves of *Tradescantia virginiana* (Rezaei Nejad and van Meeteren, 2007), *Vicia faba* (Aliniaeifard et al., 2014), and *Rosa × hybrida* (Arve et al., 2013; Giday et al., 2013) developed at high RH (90%) compared to those developed at moderate RH (60%). The active hormone concentration in the tissue depends on its biosynthetic and catabolic rates (Nambara and Marion-Poll, 2005). The major cause of inactivation of free ABA is oxidation. Free ABA is firstly hydroxylated to PA, which is reduced to DPA (Cutler and Krochko, 1999; Nambara and Marion-Poll, 2005). Free ABA is also inactivated by covalent conjugation with monosaccharides, e.g., ABA-GE, which is hypothesized to be a storage form of ABA (Cutler and Krochko, 1999). It has been shown in roses that ABA-GE is converted to free ABA during the night inducing stomatal closure (Arve et al., 2013). High RH has been found to reduce the ABA availability by inactivating the ABA to PA in *Arabidopsis thaliana* (Okamoto et al., 2009). Moreover, at high RH the ABA-GE levels remain high during the night indicating that the conversion to free ABA does not occur (Arve et al., 2013).

In higher plants, a thigmomorphogenic response (i.e., touch-induced morphological change; e.g., wind and rain) is a slow, intensity-dependent, and saturating systemic response, that translocates from the stimulated plant regions to the non-disturbed distal regions (Jaffe, 1976; Beryl and Mitchell, 1977; Erner et al., 1980). Wind is an environmental factor having several effects on plants (Grace, 1977; Ennos, 1997), depending on leaf characteristics and on its speed (Schuepp, 1993; Lambers et al., 2008). These effects include a reduction of the boundary layer thickness enhancing gas diffusion (CO_2_ and H_2_O; Schuepp, 1993; Lambers et al., 2008). Moreover, wind flow exerts drag forces causing mechanical stress on plants (Anten et al., 2010) and high wind speed has been suggested to have a positive effect on the endogenous [ABA] reducing stomatal aperture (Whitehead, 1962; Weyers and Hillman, 1979), although this effect has not yet been quantified. To the best of our knowledge, the combined effects of high MOV and high RH on plant growth and development as well as on stomatal functioning have not yet been properly explored. One of the few studies that investigated the effect of MOV (0.08, 0.21, and 0.35 m s^-1^) combined with RH (70 and 90%) found that increasing wind speed at high RH had little effect on water loss of detached leaves of cut rose (Mortensen and Gislerød, 1997). However, the air speed levels used in that study were relatively low.

Several studies have suggested that stomatal malfunctioning in high RH-grown plants is strongly related to a long-term low [ABA] during leaf development as a short-term exogenous ABA application did not increase stomatal functionality (Rezaei Nejad and Van Meeteren, 2008; Fanourakis et al., 2011). However, in those studies exogenous ABA was applied only on fully developed leaves. In rose plants it was shown that after full leaf expansion stomatal function is no longer affected either by RH (when plants were moved from high to moderate RH) or ABA levels (Fanourakis et al., 2011). Thus, it remains unclear whether the lack of stomatal responsiveness to short-term ABA feeding is due to leaf developmental stage or due to the duration of this stimulus.

In this study we aimed at understanding the physiological effect of MOV on stomatal functioning in plants developed under high RH and whether the stage of leaflet development has an influence on stomatal sensitivity to ABA. It is our hypothesis that: (1) high MOV applied 24 h per day during the whole period of leaf development increases [ABA] improving stomatal closure in high RH-grown plants; and (2) non-fully developed leaflets close better their stomata than fully developed ones in response to exogenous ABA application. Additionally, we aimed at studying the combined effect of high MOV and high RH on plant growth and visual quality parameters.

## Materials and Methods

### Plant Material and Growth Conditions

Rooted cuttings of pot rose cultivar ‘Toril’ (*Rosa* × *hybrida*) were planted in 12 cm (0.66 l) pots containing a standard fertilized *Sphagnum* peat (Floralux, Nittedal, Norway). When the broken buds were 1–1.5 cm long, 56 plants were randomly distributed over four climate controlled growth cabinets (length × width × height = 1.5 m × 1.0 m × 2.2 m). Plants were grown as single shoot, one plant per pot. During the cultivation period, the RH was 61 ± 3% (moderate RH) in two growth cabinets and 92 ± 2% (high RH) in the other two. In two growth cabinets (one per RH level), two fans (HT – 112 E, Honeywell, Lausanne, Switzerland) were located equidistant (70 cm) from the 14 plants that were placed in a semi-circle side by side, and were on during 24 h per day. Plants were rotated 90° daily in the horizontal plane to ensure that exposure to high MOV was similar in all directions (Anten et al., 2010). In the cabinets without fans (i.e., with no additional MOV) the plants were distributed similarly and were also rotated. An ultrasonic anemometer (Ultrasonic anemometer, Model 81000, Young, Traverse City, MI, USA) registered automatically the three dimensional air velocity and turbulence intensity (i.e., the percentage value calculated as the standard deviation of the fluctuations of the air velocity divided by the mean wind velocity) at plant level. Although traditionally the air speed is measured as the air flow in a specified direction (Downs and Krizek, 1997), in a closed environment, such as the one used in this study, the deflections of a high MOV on the cabinet’s wall amplify the MOV making the flow regime more turbulent than in the open field. Thus, under such environment, turbulence intensity should also be quantified. In our study, we guaranteed that plants were subjected to a strong mechanical stimuli because in addition to their visible continuous strong waving, the measured turbulence intensity ranged between 92 and 240% with an average of 142%, which is 5.7-fold higher compared to that registered in standard growth cabinets (Downs and Krizek, 1997). The air velocity in the high MOV-treated plants was 0.92 ± 0.03 m s^-1^, being 2.6-fold higher as compared to the maximum level applied by Mortensen and Gislerød (1997). In the absence of additional MOV the measured air velocity at plant level was 0.08 m s^-1^. Temperature was 21 ± 0.5°C (day and night), resulting in VPD of 0.97 ± 0.03 kPa (moderate RH) and 0.20 ± 0.01 kPa (high RH). The CO_2_ concentration was 400 ± 50 μmol mol^-1^ and high pressure sodium lamps (Plantastar 400W, Osram, Münich, Germany) provided 20 h photoperiod of 160 ± 10 μmol m^-2^ s^-1^ photosynthetic active radiation (Li-250 Light Meter, LI-COR, Lincoln, NE, USA). Climate data were recorded automatically every 5 min (Priva, De Lier, The Netherlands). Plants were watered daily until draining with a nutrient solution (Arve et al., 2013). The pH and EC levels of the nutrient solution were 5.7 and 1.75 dS m^-1^, respectively.

### Plant Growth and Plant Transpiration Rate

The effects of MOV and RH on plant growth and visual quality parameters were evaluated in fully developed plants (i.e., flower bud with cylindrical shape and pointed tip). Total plant dry weight (stem, leaves, and flower), leaf area, plant height, number of internodes, average internode length, peduncle length and diameter, flower dry weight and time to flowering (number of days from planting till full developed plant) were assessed in fourteen plants per treatment. Moreover, plant transpiration rate during the light and the dark periods were measured gravimetrically during three consecutive days using fully developed plants. Plants were watered until container capacity and pots were wrapped into impermeable plastic bags to avoid evaporation from the substrate. During this period the weight of seven plants per treatment was recorded at the beginning of the light and dark periods (Model PG503DR Delta Range, Mettler-Toledo, Greifensee, Switzerland). At the end of the 3-days period total leaf area per plant was measured using a leaf area meter (Model 3100 Area Meter, LI-COR, Lincoln, NE, USA) to calculate transpiration rate per unit leaf area.

### Stomatal Characteristics and Leaf Surface Morphology

Stomatal density, index, length, width, pore length and pore aperture were analyzed in one of the two uppermost lateral leaflets from the first fully expanded penta-foliated leaf. Epidermal impressions were made by Suzuki’s Universal Micro-Printing (SUMP) method using SUMP liquid 1 and SUMP plate B (SUMP Laboratory, Tokyo, Japan) as described by Tanaka et al. (2005). Samples were taken from the abaxial side of intact leaflets, midway between the tip and the base, away from the edge and avoiding veins, 4 h after the light period started. The imprints were observed under a light microscope (Eclipse 55i, Nikon, Tokyo, Japan) and stomatal images were obtained with a 5.24 megapixel camera (DS-Fi1, Nikon, Tokyo, Japan). To quantify stomatal density and index a magnification of 100× was used and 70 images per treatment were analyzed. The stomatal index was calculated according to Eq. 1 (Salisbury, 1927).

(1) $$Stomatal index=\frac{stomatal density}{stomatal density + epidermal cell density}\;\times \;100$$

To measure stomatal and pore size, a magnification of 400× was used and 140 stomata per treatment were evaluated. Image analysis was performed using the UTHSCSA ImageTool for windows version 3.00 (The University of Texas Health Science Center at San Antonio, San Antonio, TX, USA).

To study leaf surface morphology (namely stomatal deepness, i.e., the deepness of stomatal insertion in the leaf epidermis, and leaf epidermal cells shape/undulation) 0.5 cm × 0.5 cm leaf sections excised close to the midrib, midway between the tip and the base, away from the edge and avoiding veins were observed under a scanning electron microscope (Zeiss EVO – 50 – EP, Carl Zeiss SMT Ltd., Cambridge, UK). Samples were fixed in 1.25% glutaraldehyde and 2% paraformaldehyde in 0.05 M PIPES buffer, pH 7.2, and kept in PIPES buffer (0.1 M, pH 7). After fixation, samples were dried by the use of a critical point dryer (Bal-Tec CPD 030, Bal-Tec AG, Balzers, Germany) with dehydration series of 70, 90, 96, and 100% ethanol. Samples were mounted on aluminum stubs and coated in a sputter coater (Polaron SC 7640, Quorum Technologies Ltd., Ringmer, East Sussex, UK). Four biological replicates per treatment were analyzed.

### Stomatal Responsiveness to Leaflet Desiccation

Stomatal responsiveness to leaflet desiccation (i.e., stomatal closing stimulus) was evaluated by determining the transpiration rate and RWC in detached terminal leaflets. Determination of the transpiration rate by gravimetry is an adequate quantitative description of the stomatal functionality as demonstrated by Rezaei Nejad and van Meeteren (2005). This simple procedure has been thoroughly used in this type of studies (Rezaei Nejad and van Meeteren, 2005; Giday et al., 2013; Fanourakis et al., 2015) due to its effectiveness under conditions of low stomatal conductance, e.g., excessively desiccated leaflets (i.e., RWC < 20%), which fall below the detection limit of the porometer. Fully developed leaflets (first penta-foliated, counting from the apex) were detached from the plants and their petioles were recut under MilliQ-water to avoid cavitation-induced embolism. To establish leaflet saturated fresh weight, leaflets were placed with their petioles in a vial with MilliQ-water and were incubated in light (11.2 ± 0.2 μmol m^-2^ s^-1^; Philips TL 58W, color 84) for 1 h at about 100% RH (23.7 ± 1.3°C; VPD close to 0; Fanourakis et al., 2011). Because leaflets were detached from the plants at the beginning of the light period, the rehydration was also conducted in light, since following darkness the light-induced stomatal opening might require up to 1 h (Blom-Zandstra et al., 1995; Drake et al., 2013). After rehydration, petioles were removed from the water and leaflets were allowed to desiccate under constant conditions [abaxial surface down; 1.68 kPa VPD (42.7 ± 7.3% RH, 23.7 ± 1.3°C) and 11.2 ± 0.2 μmol m^-2^ s^-1^ light intensity]. Leaflets were weighted every 5–30 min for 4 h. Leaflet area, dry weight (24 h at 70°C) and transpiration rate were determined, and RWC was calculated using Eq. 2 (Slavík, 1974). One leaflet per plant was evaluated (14 plants per treatment).

(2) $$\mathrm{RWC}\;=\;\frac{\mathrm{fresh}\mathrm{weight}-\mathrm{dry}\mathrm{weight}}{\mathrm{saturated}\mathrm{fresh}\mathrm{weight}-\mathrm{dry}\mathrm{weight}}\;\times \;100$$

### Endogenous ABA Quantification

Fully developed tri-foliated leaves (just above the first penta-foliated) were sampled 5 h after the beginning of the light period, immediately frozen in liquid nitrogen and stored at -80°C till analysis. Two composite samples (each with seven biological replicates) per treatment were evaluated.

#### Chemicals and Calibration Curves

Standard ABA-catabolites (PA, DPA, ABA-GE, 7′-OH-ABA, neoPA, and *trans*-ABA), deuterated forms of ABA-catabolites used as internal standards (i.e., d3-DPA, d5-ABA-GE, d3-PA, d4-7′-OH-ABA, d3-neoPA, d4-ABA, and d4-*trans*-ABA) according to Abrams et al. (2003) and Zaharia et al. (2005) and the deuterated forms of the selected compounds used as recovery standards (i.e., d6-ABA and d2-ABA-GE), were synthesized and prepared at the National Research Council of Canada (NRCC, Saskatoon, SK, Canada). *Cis*-ABA was purchased from Sigma-Aldrich (Sigma Chemicals, St. Louis, MO, USA).

#### Extraction and Purification

Samples were freeze dried and homogenized before analysis. A 100 μL aliquot containing the deuterated internal standards, each at a concentration of 0.2 pg μL^-1^, was added to ∼50 mg of homogenized plant tissue; 3 mL of isopropanol:water:glacial acetic acid (80:19:1, v/v/v) was added and the samples were agitated in the dark for 24 h at 4°C. Samples were then centrifuged and the supernatant was isolated and dried on a Büchi Syncore Polyvap (Büchi, Flawil, Switzerland). Samples were reconstituted in 100 μL acidified methanol, adjusted to 1 mL with acidified water, and then partitioned against 2 mL hexane. After 30 min, the aqueous layer was isolated and dried as above. Dry samples were reconstituted in 100 μL acidified methanol and adjusted to 1 mL with acidified water. The reconstituted samples were loaded onto equilibrated Oasis HLB cartridges (Waters, Mississauga, ON, Canada), washed with acidified water and eluted with acetonitrile:water:glacial acetic acid (30:69:1, v/v/v). The eluate was then dried on a LABCONCO centrivap concentrator (Labconco Corporation, Kansas City, MO, USA). An internal standard blank was prepared with 100 μL of the deuterated internal standards mixture. Quality control (QC) standards were prepared by adding 100 and 30 μL (separately) of a mixture containing the analytes of interest, each at a concentration of 0.2 pg μL^-1^ to 100 μL of the internal standard mix. Finally, samples, blanks, and QCs were reconstituted in an aqueous solution of 40% methanol (v/v), containing 0.5% acetic acid and 0.1 pg μL^-1^ of each of the recovery standards.

#### Hormone Quantification by HPLC-ESI-MS/MS

The samples were subjected to HPLC-ESI-MS/MS analysis and quantification (Ross et al., 2004). Samples were injected onto an ACQUITY UPLC HSS C18 column (2.1 mm × 100 mm, 1.8 μm) with an ACQUITY HSS C18 VanGuard Pre-column (2.1 mm × 5 mm, 1.8 μm) and separated by a gradient elution of water containing 0.025% acetic acid against an increasing percentage of acetonitrile containing 0.025% acetic acid. Briefly, the analysis utilizes the multiple reaction monitoring (MRM) function of the MassLynx v4.1 (Waters, Inc.) control software. The resulting chromatographic traces are quantified off-line by the QuanLynx v4.1 software (Waters, Inc.) wherein each trace is integrated and the resulting ratio of signals (non-deuterated/internal standard) is compared with a previously constructed calibration curve to yield the amount of analyte present (ng per sample). Calibration curves were generated from the MRM signals obtained from standard solutions based on the ratio of the chromatographic peak area for each analyte to that of the corresponding internal standard, as described by Ross et al. (2004). The QC samples, internal standard blanks and solvent blanks were also prepared and analyzed along each batch of tissue samples.

### Stomatal Responsiveness to ABA Feeding through the Transpiration Stream

Stomatal responsiveness to exogenous ABA feeding (i.e., stomatal closing stimulus) through the transpiration stream was evaluated in detached terminal leaflets in two stages of expansion: 100% FLE and 70–80% FLE. The percentage of FLE was defined as the proportion of leaflet length at harvest relative to its final length (i.e., when the midrib stopped elongating for three consecutive days; Fanourakis et al., 2011). Terminal leaflets from the second penta-foliated leaves were used as 100% FLE samples. The developmental stage of the terminal leaflet from the third penta-foliated leaf (intended to be 70–80% of the FLE) was estimated based on its length and the elongation curve of the terminal leaflet from the fourth penta-foliated leaf, of which its length was recorded daily from unfolding till 100% FLE. Leaflet detachment and rehydration were conducted as described above for evaluation of the stomatal responsiveness to desiccation. After 30 min of rehydration in vials with MilliQ-water, leaflets were transferred to a vial with 0 or 100 μM (±) ABA solution (Sigma, St. Louis, MO, USA) and were weighted every 5–10 min during 150 min. At the end, leaflet area was measured and transpiration rate was calculated. ABA intake was calculated as the product of leaflet transpiration rate and the concentration of the feeding solution (Fanourakis et al., 2013). The changes in transpiration rate in response to ABA showed the features of a dose-response curve (ABA intake was considered as the dose), and was fitted with a four parameter logistic model as described by Giday et al. (2013). The model fitting (Eq. 3) was performed using GraphPad Prism (version 6.00 for Windows, GraphPad Software, San Diego, CA, USA).

(3) $$Transpiration rate=minimum value+\frac{(maximum value-minimum value)}{(1+10^{\wedge }((LogEC50-ABA intake)\times hill slope)}$$

In Eq. 3, the coefficients maximum and minimum values correspond to the transpiration rate before (*t* = 0) and after (*t* = 2.5 h) ABA feeding, respectively. EC_50_ describes the amount of ABA required to reduce the transpiration half-way between its maximum and minimum values. Hill slope represents the steepness of the curve. One leaflet per plant was evaluated from five or six plants per treatment.

### Statistical Design and Analysis

The experimental set-up was a 2 by 2 factorial design and the experiment was repeated once. Analysis of variance was conducted, considering individual plants as experimental units. Main effects and interactions were tested at *P* = 0.05. When relevant, Fisher’s least significant difference (LSD) at *P* = 0.05 was calculated to separate interaction means. The Genstat software (15th Edition; VSN International Ltd., Herts, UK) was used for the analysis.

## Results

### Plant Growth, Visual Quality, and Plant Transpiration Rate

The only significant effect of MOV on plant growth and visual quality parameters was a 6% increase on peduncle diameter (*P* = 0.022; **Table 1**). High RH during growth did not significantly affect total dry weight (*P* = 0.174), total leaf area (*P* = 0.446), number of internodes (*P* = 0.250), or time to flowering (*P* = 0.480). However, it significantly increased plant height (*P* < 0.001), resulting in 9% taller plants, longer peduncle length (12%; *P* < 0.001) and higher average internode length (6%; *P* = 0.010; **Table 1**). Additionally, flower dry weight and peduncle diameter were significantly reduced in high RH-grown plants: 13% (*P* = 0.002) and 12% (*P* < 0.001), respectively (**Table 1**).

**Table 1:** Plant growth and visual quality parameters in fully developed plants (i.e., flower bud with cylindrical shape and pointed tip) of pot rose cv. Toril grown at moderate (61%) or high (92%) RH without (-MOV) or with (+MOV) additional MOV.

	RH	-MOV	+MOV	Mean
Total dry weight (g)	61%	6.4	6.5	6.5
	92%	6.9	7.0	6.9
	Mean	6.6	6.8	
Total leaf area (cm^2^)	61%	545.9	572.5	559.2
	92%	538.6	535.8	537.2
	Mean	542.2	554.1	
Plant height (cm)	61%	34.3	35.2	34.8^a^
	92%	37.3	38.2	37.8^b^
	Mean	35.8	36.7	
Number of internodes	61%	11.7	11.9	11.8
	92%	11.9	12.6	12.3
	Mean	11.8	12.3	
Average internode length (cm)	61%	2.2	2.2	2.2^a^
	92%	2.4	2.3	2.3^b^
	Mean	2.3	2.3	
Peduncle length (cm)	61%	5.4	5.4	5.4^a^
	92%	6.0	6.1	6.1^b^
	Mean	5.7	5.8	
Peduncle diameter (mm)	61%	4.1	4.5	4.3^b^
	92%	3.7	3.8	3.8^a^
	Mean	3.9^a^	4.2^b^	
Flower dry weight (g)	61%	1.8	2.0	1.9^b^
	92%	1.7	1.7	1.7^a^
	Mean	1.8	1.9	
Time to flowering (days)	61%	31.4	31.3	31.4
	92%	30.5	31.6	31.1
	Mean	31.0	31.5	

Different letters indicate significant differences according to Fisher’s LSD test (*P* = 0.05).

**FIGURE 1:**
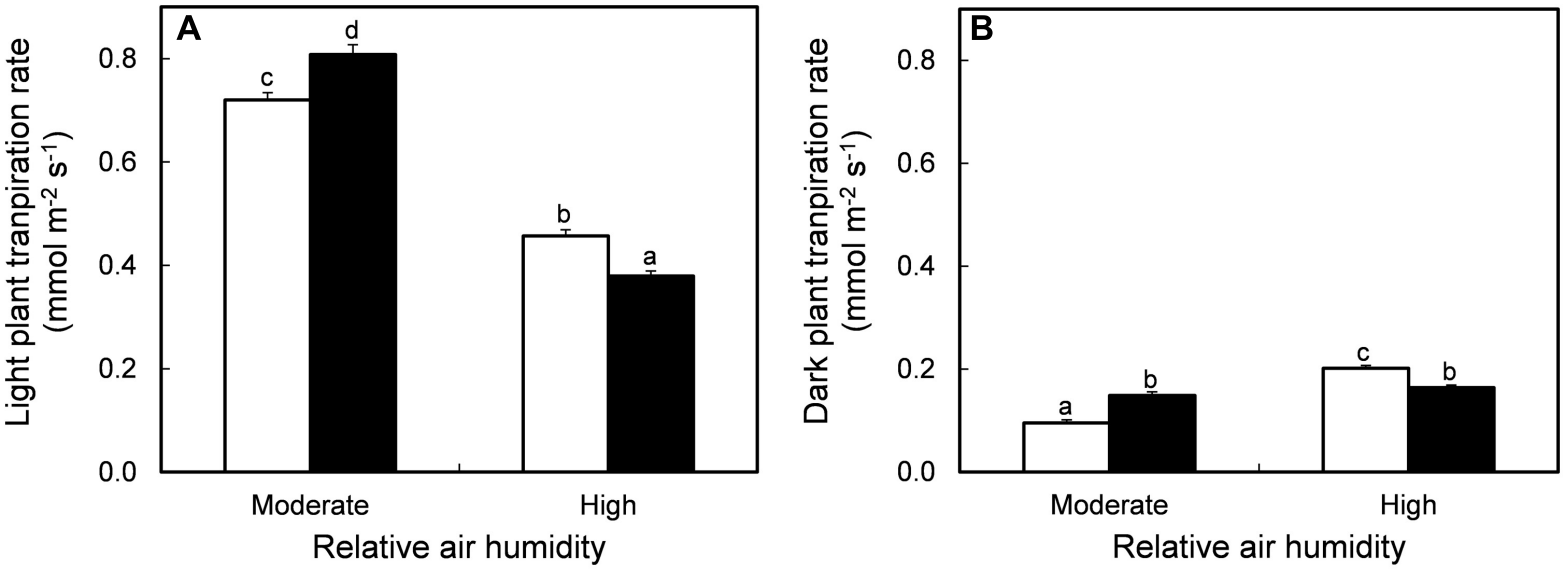
**Transpiration rate in intact plants during light **(A)** and dark **(B)** period in pot rose cv. ‘Toril’ grown at moderate (61%) or high (92%) RH, without (open columns) and with (solid columns) additional MOV.** Measurements were conducted throughout three consecutive days in fully grown plants, starting when the flower bud had cylindrical shape and pointed tip. Values are the mean of 14 intact plants and bars represent the SEM. Different letters indicate significant differences according to Fisher’s LSD test (*P* = 0.05).

The effect of MOV on plant transpiration rate at growth conditions depended on the RH level (**Figure 1**). At growth conditions during the light period, high MOV increased the transpiration rate by 12% in intact plants grown at moderate RH, while it was decreased by 17% in high RH-grown plants (*P* < 0.001; **Figure 1A**). The same trend was observed in the dark period where high MOV increased the transpiration rate by 57% in moderate RH-grown plants, whereas the transpiration rate was 19% lower in high RH-grown plants (*P* < 0.001; **Figure 1B**). In all treatments, darkness led to a lower transpiration rate, but this reduction was stronger in plants grown at moderate (84%) than at high RH (56%). Moreover, there was no MOV effect on the transpiration rate reduction in response to darkness. These results indicate that stomatal response to darkness is lower in high RH-grown plants compared to moderate RH-grown plants and that high MOV did not improve the dark-induced stomatal closure.

### Stomatal Physiology and Morphology

**FIGURE 2:**
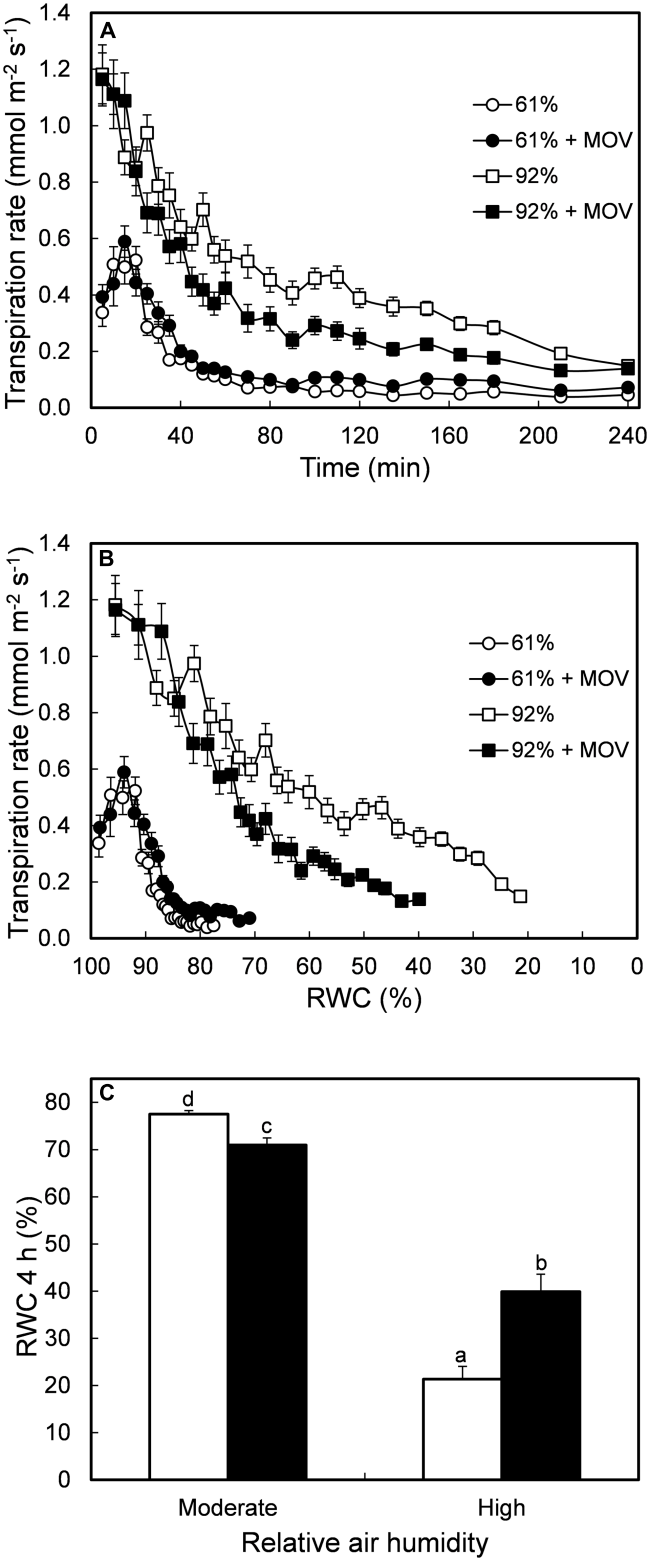
**Transpiration rate as a function of time of desiccation **(A)** and as a function of RWC **(B)** during 4 h of leaflet desiccation.** RWC after 4 h of leaflet desiccation **(C)**. All measurements were conducted in pot rose cv. ‘Toril’ grown at moderate (61%; circles) or high (92%; squares) RH, without (open symbols) or with (solid symbols) additional MOV. Values are the mean of 28 detached leaflets and bars represent SEM. Different letters indicate significant differences according to Fisher’s LSD test (*P* = 0.05).

Desiccated leaves from moderate RH-grown plants showed lower transpiration rates, irrespective of MOV, when compared to leaflets from plants developed at high RH (**Figure 2A**). Leaflets grown under high RH with or without additional MOV had a similar initial transpiration rate, but stomata from high MOV leaflets closed faster in response to leaf desiccation (**Figure 2B**). This resulted in a RWC after 4 h of desiccation twofold higher in high RH-grown plants with high MOV as compared to high RH-grown plants without additional MOV (**Figure 2C**).

High MOV reduced the pore aperture by 16% (*P* = 0.002) and the pore length by 6% (*P* = 0.022) in stomata developed at high RH, while there was no significant effect on stomata developed at moderate RH (**Table 2**). Moreover, MOV had no significant effect on stomatal density (*P* = 0.060), index (*P* = 0.719), length (*P* = 0.189) and width (*P* = 0.970), but increasing the RH significantly increased these features by 4, 13, 20, and 26% (*P* < 0.001), respectively (**Table 2**).

**Table 2:** Stomatal characteristics of pot rose cv. ‘Toril’ grown at moderate (61%) or high (92%) RH, without (-MOV) or with (+MOV) additional MOV in intact fully expanded leaves, 4 h after the beginning of the light period.

	Moderate RH			High RH		
	-MOV	+MOV	Mean	-MOV	+MOV	Mean
Stomatal density (no mm^-2^)	57.2	55.8	56.5^a^	59.8	57.8	58.8^b^
Stomatal index (%)	12.4	12.4	12.4^a^	13.9	14.0	14.0^b^
Stomatal length (μm)	27.4	26.8	27.1^a^	32.6	32.4	32.5^b^
Stomatal width (μm)	15.4	15.5	15.4^a^	19.6	19.5	19.5^b^
Pore length (μm)	17.3^a^	17.2^a^	17.2	24.5^c^	23.1^b^	23.8
Pore aperture (μm)	3.4^a^	3.2^a^	3.3	6.8^c^	5.7^b^	6.2

Values are the mean of 70 field views (stomatal density and index) and 140 stomata (stomatal length and width, pore length and aperture). Different letters represent significant differences according to Fisher’s LSD test (*P* = 0.05; comparison in rows).

**FIGURE 3:**
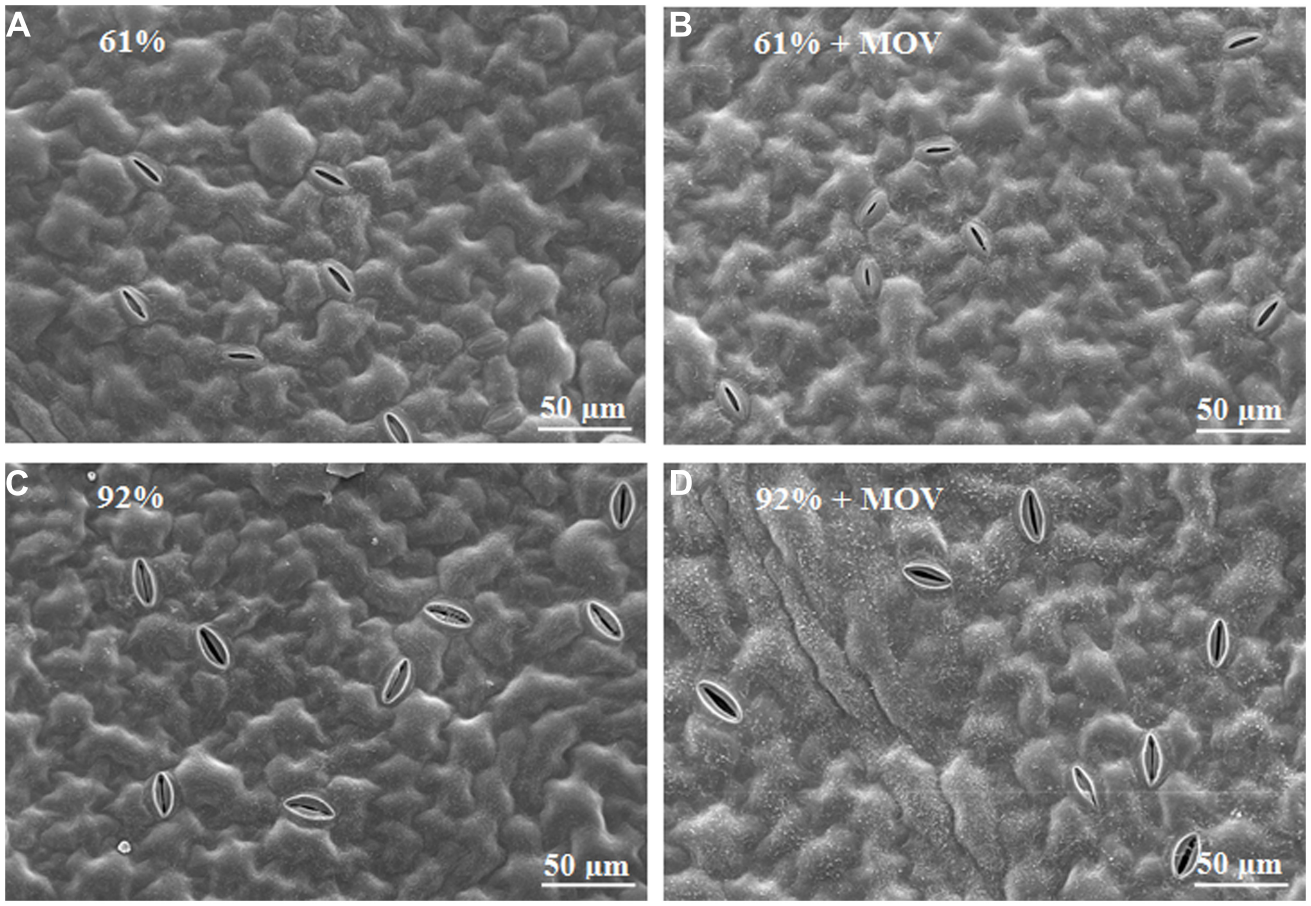
**Morphology of the abaxial leaf surface in pot rose cv. ‘Toril’ grown at moderate (61%; **A,B**) or high (92%; **C,D**) RH combined with no additional MOV **(A,C)** or with additional MOV (+MOV; **B,D**).** Images were obtained by scanning electron microscope.

Exposure to high MOV did not induce visual morphological changes on the leaf epidermal cells (e.g., shape or undulations) neither on the adaxial (data not shown) nor on the abaxial surfaces (**Figure 3**). Additionally, high MOV did not visually affect stomatal deepness i.e., how deep the stomata are inserted in the leaf epidermis. However, a clear increase in stomatal size at high RH, already described above, was also apparent when analyzing leaf surface using the scanning electron microscope.

### Stomatal Responsiveness to ABA

**FIGURE 4:**
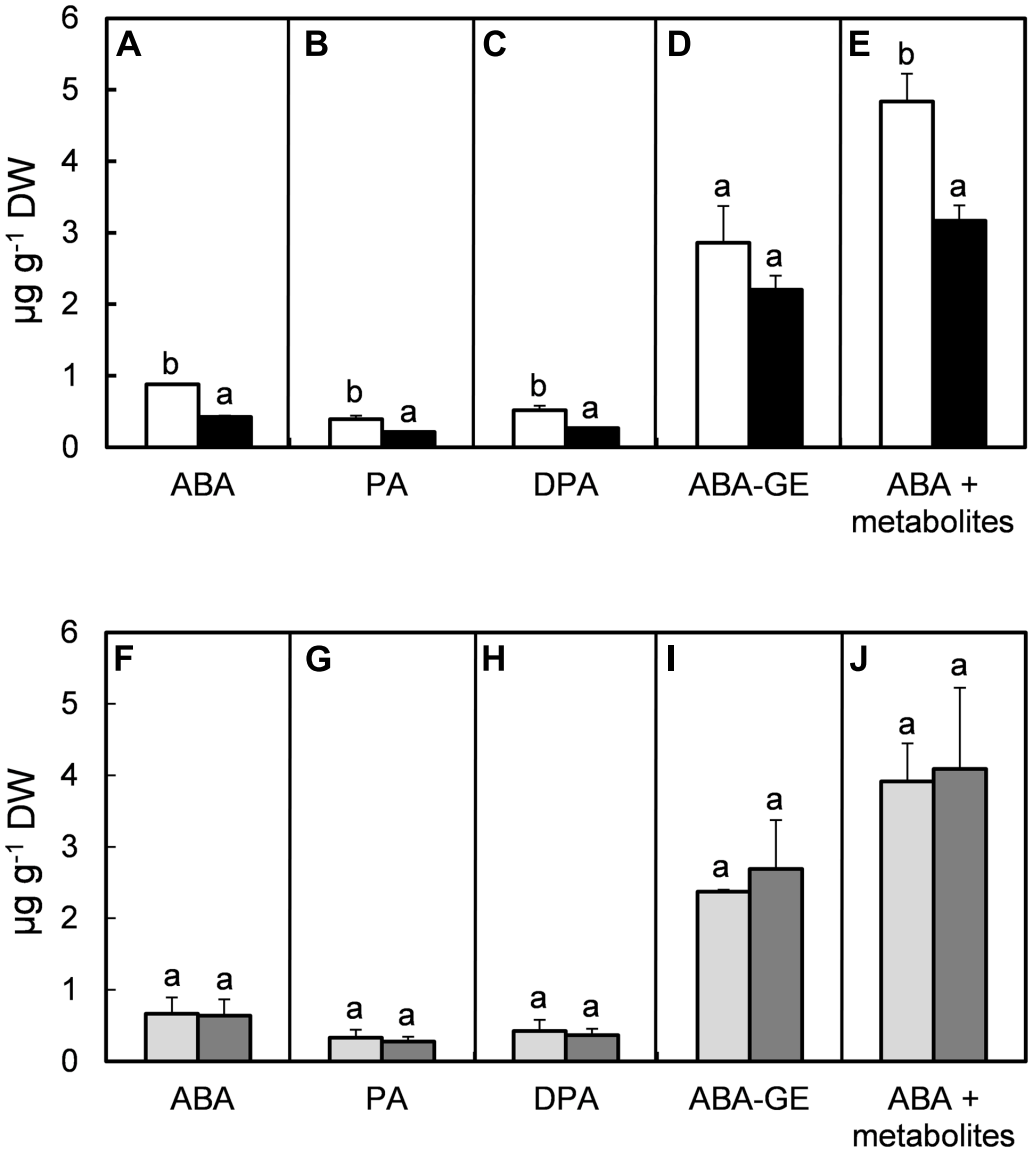
**Effect of moderate (61%; open columns) and high (92%; solid columns) RH **(A–E)**, combined with no additional MOV (light gray columns) or with additional MOV (gray columns; **F–J**) on the levels (μg g^**-1**^ DW) of ABA **(A,F)**, PA **(B,G)**, DPA **(C,H)**, ABA-GE **(D,I)** and total concentration of ABA and its metabolites **(E,J)** in leaves of rose plants cv. ‘Toril.’ Sampling occurred 5 h after the beginning of the light period.** Each sample consisted of a composite of seven leaflets from seven biological replicates. Values are the mean of two biological repeats and bars represent the SEM. Different letters indicate significant differences according to Fisher’s LSD test (*P* = 0.05).

Abscisic acid and its metabolites (PA, DPA, ABA-GE, 7′OH-ABA, *neo*PA, *trans*-ABA, and *cis-*ABA) were quantified in fully developed leaves. In all treatments, the levels of 7′OH-ABA, *neo*PA, *trans*-ABA, and *cis-*ABA were very low (data not shown), hence, they have only been included in the combined amount of ABA and its metabolites when quantification was possible. High RH reduced the concentrations of ABA by 52% (*P* = 0.005; **Figure 4A**), PA by 46% (*P* = 0.008; **Figure 4B**), DPA by 48% (*P* = 0.004; **Figure 4C**), ABA-GE by 23% (*P* = 0.184; **Figure 4D**) and the combination of ABA and its metabolites by 35% (*P* = 0.013; **Figure 4E**). High MOV did not significantly affect the concentration of ABA (*P* = 0.764, **Figure 4F**), PA (*P* = 0.224, **Figure 4G**), DPA (*P* = 0.234, **Figure 4H**), ABA-GE (*P* = 0.488, **Figure 4I**), or combined ABA and its metabolites (*P* = 0.671, **Figure 4J**).

In fully developed leaflets (100% FLE) there was a significant interaction between RH and MOV (*P* = 0.028; **Table 3**). When these leaves were grown at high RH, high MOV reduced by 48% the EC_50_, while it was not significantly affected in moderate RH-grown plants. In non-fully developed leaflets (70–80% FLE) grown at high RH without MOV stomatal response to ABA feeding was practically absent (i.e., the stomatal remained open with no reduction in the transpiration rate), making it impossible to determine the EC_50_ (**Table 3**). However, high RH-grown plants with high MOV showed an EC_50_ of 0.431, which was about 2.3 times higher than the one observed in moderate RH-grown plants (**Table 3**). In moderate RH-grown plants, MOV did not affect EC_50_ (**Table 3**). EC_50_ was not significantly different when comparing non-fully developed leaflets with fully developed leaflets (moderate RH without MOV, *P* = 0.060; moderate RH with high MOV, *P* = 0.064; high RH with MOV, *P* = 0.592; **Table 3**).

**Table 3:** Abscisic acid intake required to reduce the transpiration rate to half-way (50%) between the maximum and minimum values (EC_**50**_) in fully developed leaflets (100% FLE) and non-fully developed leaflets (70–80% FLE) of pot rose cv. Toril grown at moderate (61%) or high (92%) RH, without (-MOV) or with (+MOV) additional MOV.

Leaf developmental stage (% FLE)		Moderate RH	High RH
100%	-MOV	0.121^a^	0.758^c^
	+MOV	0.106^a^	0.395^b^
70–80%	-MOV	0.212^a^	^∗^
	+MOV	0.169^a^	0.431^b^

Abscisic acid feeding (100 μM) through the leaflet petiole lasted for 150 min. Values are the mean of five detached leaflets. Different letters indicate significant differences according to Fisher’s LSD test (*P* = 0.05; comparison within leaf developmental stage).

*In high RH (92%) without additional MOV the stomatal response to ABA was practically absent, making it impossible to determine the EC_50_.

## Discussion

### Effects of MOV and RH on Plant Transpiration Rate

It is well-known that high wind-speed reduces leaf boundary layer, which results in enhanced transpiration rate (Schuepp, 1993; Lambers et al., 2008). Thus, the increased transpiration rate found in intact plants grown under moderate RH and subjected to high MOV can possibly be explained by the reduction of the leaf boundary layer (Mortensen and Gislerød, 1997; Anten et al., 2010). Nevertheless, at high RH the saturated air present on the leaf boundary layer (nearly 100% RH) was replaced also by very moist air existing in the growth cabinets (92 ± 2%), which explains why high MOV did not increase the transpiration rate also under high RH levels (Mortensen and Gislerød, 1997). Actually, in these plants, the lower stomatal pore dimensions (**Table 2**) might have contributed to their reduced plants transpiration rate (**Figure 1**), via a reduction in the total transpiration area.

### Effects of MOV and RH on the Stomatal Responsiveness to Closing Stimuli

In *Arabidopsis* the cuticle wax deposition contributes to enhance the water loss tolerance (Seo et al., 2011; Yang et al., 2011; Zhu et al., 2014). However, in *R. hybrida*, the cuticle has a minor contribution to the total leaf water loss while an increase in the leaf transpiration rate seems to largely reflect a higher stomatal pore area per leaf area (Fanourakis et al., 2013). Long-term high RH is known to decrease stomatal responsiveness to closing stimuli leading to high transpiration rate and lower RWC upon desiccation (Torre et al., 2003; Rezaei Nejad and van Meeteren, 2005) and darkness (Arve et al., 2013; Fanourakis et al., 2013). Our results confirm those findings (**Figures 1** and **2**) and demonstrated for the first time that MOV is effective in increasing stomatal responsiveness to desiccation in high RH-grown leaflets, resulting in a twofold higher RWC as compared to leaflets without additional MOV (**Figure 2C**). In spite of the improved stomatal functioning in high RH-grown leaflets subjected to MOV compared to still air, the RWC of the former was still far below the one of moderate RH-grown plants (**Figures 2A,B**). This can be partly explained by their initial higher transpiration rate contributing to a large water loss in the first phase of leaflet desiccation, before the stomata trigger the closure response.

Abscisic acid is a very important hormone inducing stomatal closure under different abiotic stress conditions (Xiong et al., 2002; Davies et al., 2005; Schachtman and Goodger, 2008). Unlike our hypothesis, it was found that despite the increased stomatal responsiveness to desiccation in high RH-grown plants subjected to high MOV (**Figure 2**), this did not significantly increase the endogenous [ABA] and its metabolites in the bulk leaves (**Figures 4F–J**). Other reasons such as (1) perception and/or sensitivity of ABA receptors (Anderson et al., 1994; Schwartz et al., 1994) which may differ in fully expanded and in expanding leaves and (2) [ABA] in the guard cells (Harris and Outlaw, 1991) might also be involved in stomatal closure. High MOV decreased the required amount of exogenous ABA to reduce in 50% the half-maximal effective concentration (EC_50_) in detached fully expanded leaflets grown at high RH (**Table 3**). Although in *Arabidopsis*, Aliniaeifard and van Meeteren (2014) did not find a correlation between stomatal responsiveness to desiccation and stomatal sensitivity to ABA, our results suggest that in high RH-grown plants, high MOV increased stomatal tolerance to desiccation due to increased stomatal sensitivity to ABA, rather than an increase in the leaf [ABA]. Pantin et al. (2013) suggested that stomatal sensitivity to ABA is related to the leaf developmental stage in *A. thaliana*. Here we found no difference in the stomatal responsiveness to ABA feeding between leaf developmental stages (**Table 3**) indicating that even non-fully mature stomata grown at high RH do not respond to a short-term ABA application.

Concerning the effect of high RH on the free [ABA], ‘Toril’ reduced by 35% the concentration of ABA and its metabolites (**Figure 4E**). These findings are in agreement with Giday et al. (2013) who found a 25–35% decrease in the [ABA] in the sensitive cultivars. Similarly, the concentrations of the metabolites PA (**Figure 4B**) and DPA (**Figure 4C**) followed the pattern of ABA (**Figure 4A**). Arve et al. (2013) described the same tendency and suggested that low PA and DPA levels seem to be a result of low ABA levels at high RH and a constant inactivation rate of ABA to PA and consequently to DPA.

### Effects of MOV and RH on Stomatal Anatomy and Plant Morphology

The absence of high MOV effect in most of the stomatal anatomical features and in the leaf ultrastructure (**Table 2** and **Figure 3**) is in contrast with a previous study in *Picea sitchensis* and *Pinus sylvestris* (van Gardingen et al., 1991) which described modifications on the leaf surface in plants grown under high MOV (11 m s^-1^). This might indicate that different species, with contrasting leaf morphology, respond differently to MOV but it can also be due to the extremely high MOV intensity applied to those trees as compared to the MOV used in this study. Stomatal density and index were significantly increased at high RH, but according to Fanourakis et al. (2013) this effect seems to be cultivar dependent. Moreover, our study confirmed that a sensitive cultivar (‘Toril’) responds to high RH enhancing their stomatal and pore dimensions (**Table 2**), as previously reported for other sensitive rose cultivars (Torre et al., 2003; Arve et al., 2013; Fanourakis et al., 2013; Giday et al., 2013). In contrast, high RH did not affect the leaf ultrastructure (**Figure 3**) and it had only a minor effect on plant growth and visual quality parameters (**Table 1**), which is in agreement with previous studies (Mortensen and Gislerød, 1997; Torre and Fjeld, 2001; Torre et al., 2003). The thinner peduncles observed in high-RH grown plants can partly contribute to the higher incidence of bent-neck symptoms during post-harvest, which is typically found in plants developed at high RH (Fanourakis et al., 2012). Here, we show that this positive effect of MOV on peduncle diameter (**Table 1**) might partly reduce the bent-neck incidence in high RH-grown plants. Mortensen and Gislerød (1997) also reported little effect of MOV on plant height, no effect on total dry weight but an increased time to flowering. It is concluded that unlike MOV, high RH has a strong effect on stomatal anatomy in *R. hybrida*, whereas leaf surface morphology as well as plant growth and visual quality parameters are not affected by either RH of MOV, evidencing that rose plants seem to be well-adapted to high MOV.

## Conclusion

The current work shows for the first time that high MOV during leaf development improves stomatal functioning of leaves developed at high RH. Unlike our hypothesis, we have shown that this is due to an increased sensitivity to ABA (evidenced by a lower transpiration rate in leaflets fed with exogenous ABA) and reduced stomatal pore length and aperture rather than an increase in the leaf [ABA] or in the concentration of its metabolites. Finally, in this study we showed that leaf developmental stage had no effect in the stomatal responsiveness to ABA feeding demonstrating that even non-fully mature stomata grown at high RH do not respond to a short-term ABA application.

## Author Contributions

DC performed the experiments and collected the plant data. DC and DK conducted the measurements on air speed and DK executed their analysis, interpreted, wrote and edited the text about MOV. DC and EH analyzed the data. DC, ST, DA, EH, and SC interpreted the results, wrote and edited the manuscript.

## Conflict of Interest Statement

The authors declare that the research was conducted in the absence of any commercial or financial relationships that could be construed as a potential conflict of interest.

## Abbreviations

[ABA]: abscisic acid concentration
ABA: abscisic acid
ABA-GE: ABA-glucosyl ester
DPA: dihydrophaseic acid
FLE: full leaflet expansion
MOV: air movement
PA: phaseic acid
RH: relative air humidity
RWC: relative water content
VPD: vapor pressure deficit.
